# A Case of *Aeromonas trota* in an Immunocompromised Patient with Diarrhea

**DOI:** 10.3390/microorganisms8030399

**Published:** 2020-03-12

**Authors:** Ana Fernández-Bravo, Isabel Fort-Gallifa, Frederic Ballester, Isabel Pujol, Frederic Gomez-Bertomeu, Mariola Domínguez, Miquel Micó, Eva Alcoceba, Josep Maria Simó-Sisó, Maria José Figueras

**Affiliations:** 1 Unidad de Microbiología, Departamento de Ciencias Médicas Básicas, Facultad de Medicina y Ciencias de la Salud, IISPV, Universidad Rovira i Virgili, Reus 43201, Spain; ana.fernandez@urv.cat (A.F.-B.); isabel.pujol@urv.cat (I.P.); ffgomez.hj23.ics@gencat.cat (F.G.-B.); 2 Hospital Universitari Sant Joan de Reus-Laboratori de Referència de Tarragona i Terres de l’Ebre, Reus 43201, Spain; isafortgal@gmail.com (I.F.-G.); fballester1959@gmail.com (F.B.); jmsimo@lrsud.cat (J.M.S.-S.); 3 Hospital Universitari Joan XXIII-Hospital Universitari Sant Joan de Reus-Laboratori de Referència de Tarragona i Terres de l’Ebre- Facultat de Medicina i Ciències de la Salut, IISPV, Universitat Rovira i Virgili, Reus 43204, Spain; 4 Departamento de Geriatría, Hospital de la Santa Creu i Jesús, Tortosa 43590, Spain; mdominguez@saluttortosa.cat; 5 Laboratori de Referència de Catalunya, Barcelona 08820, Spain; mmicog@lrc.cat (M.M.); ealcoceba@lrc.cat (E.A.)

**Keywords:** Aeromonas trota, Aeromonas enteropelogenes, ampicillin, MALDI-TOF

## Abstract

According to recent literature, 95.4% of the *Aeromonas* strains associated with human clinical cases correspond to four species: *Aeromonas caviae*, *Aeromonas dhakensis*, *Aeromonas veronii* and *Aeromonas hydrophila.* However, other less prevalent species such as *Aeromonas trota,* are also described from clinical samples. Based on its low incidence, the latter species can be regarded as rare and it is the only *Aeromonas* species susceptible to ampicillin. From the taxonomic point of view, *A. trota* is considered a synonym of the species *Aeromonas enteropelogenes.* The objective of this study is to present a new clinical case associated with *A. trota* in order to increase the knowledge about this species. The strain was recovered from the feces of a 69-year-old patient with a diarrheal syndrome and peritoneal psammocarcinoma. The preliminary identification as *Aeromonas* sp. was obtained with the API 20E, but it was characterized as *Aeromonas jandei* and also as *Aeromonas enteropelogenes* with different scores with the matrix-assisted laser desorption ionization time of flight (MALDI-TOF). Based on the sequence of the *rpoD* gene, it was confirmed to be *A. trota*. The antimicrobial resistance pattern showed that the strain was susceptible to ampicillin, penicillins in combination with beta-lactamase inhibitors, quinolones, carbapenems, aminoglycosides and cephalosporins, except cephalothin. In conclusion, the recognition of an *Aeromonas* strain susceptible to ampicillin should alert the clinical microbiologist of the possible involvement of this rare species. Furthermore, the MALDI-TOF database should be updated indicating that the species *A. enteropelogenes*, is a synonym of *A. trota*.

## 1. Introduction

The genus *Aeromonas* includes more than 32 species, some of which are distributed in the environment and are considered autochthonous of aquatic systems [1,2,3]. *Aeromonas* spp. are considered emerging pathogens that cause a wide spectrum of diseases in humans, mainly gastroenteritis, bacteremia and wound infections, being able to infect both immunocompromised and immunocompetent patients [1,2,4]. Recent literature showed that 95.4% of the strains associated with clinical cases correspond to four species, namely *Aeromonas caviae* (37.26%), *Aeromonas dhakensis* (23.49%), *Aeromonas veronii* (21.54%) and *Aeromonas hydrophila* (13.07%) [2,5], but other less prevalent species, such as *Aeromonas trota* (0.27%)*,* are also described from clinical samples. Therefore, based on the low incidence, *A. trota* can be regarded as a rare species. This species is considered a synonym of the species *Aeromonas enteropelogenes* and both have been isolated from feces of patients with diarrhea and are the only *Aeromonas* species susceptible to ampicillin [6,7,8,9].

This study describes a case of diarrhea produced by a strain of *A. trota* and provides the results of the antimicrobial pattern determined with the MicroScan WalkAway (Siemens^®^). The isolate was first identified with API 20E and re-identified with matrix-assisted laser desorption ionization time of flight (MALDI-TOF), and on the basis of the sequences of the *rpoD* gene phylogeny [10].

## 2. Case Report

A 69-year old female with a previously peritoneal psammocarcinoma and a colostomy performed a few years ago was hospitalized in January at the emergency department of University Hospital Sant Joan de Reus in Spain, with an episode of deterioration of her general condition and abdominal pain with bleeding soft stools and without fever. In addition, her skin was pale, hydration was correct, and the abdomen examination showed normal findings. The blood test performed upon hospitalization revealed an acute renal failure with creatinine values of 4.5 mg/dL and 157 mg/dL, pH values were in the normal range, and she did not present anemia. With all the data the patient was diagnosed with diarrheal syndrome and a stool sample was collected for the analysis of bacteria, viruses and parasites. An intravenous treatment with imipenem ciprofloxacin 200 mg/100 mL every twelve hours was empirically initiated, for eleven days. After this time, the patient seemed recovered from her abdominal episode.

The analysis of viruses and parasites showed to be negative but a culture on xylose lysine deoxycholate agar (XLD) (BioMerieux ^®^, Marc l’Etoile, France) after 24 h at 37 ºC was positive. The isolate 1183C was identified as *Aeromonas* sp. based on phenotypic tests as oxidase production and the API 20E (BioMerieux^®^, Marc l’Etoile, France). Considering these results, a second identification with the MALDI-TOF Biotyper (Bruker^®^) was performed in two independent laboratories (four replicates in each laboratory) with different versions of the Biotyper database (V4 and V5) and the results are shown in Table 1. One result of MALDI-TOF showed a lower score than 2.0 (V4), and a higher score than 2.0 (V5) for *A. jandaei*, while three results presented a higher score than 2.0 (V4 and V5) for *A. enteropelogenes* (Table 1). The antibiotic susceptibility was performed with MicroScan Walkaway and the results were analyzed according to the CLSI guidelines [11]. This strain was susceptible to ampicillin and penicillin in combination with beta-lactamase inhibitors, quinolones, carbapenems, aminoglycosides and cephalosporins, with the exception of cephalothin (Table 2). The resistance pattern of the strain was compatible with *A. trota*, the only species of the genus along with its synonym *A. enteropelogenes* susceptible to ampicillin [1,2]. Similarly, the MicroScan WalkAway could not define the species, but the isolate was identified at genus level as *Aeromonas* sp.

**Figure 1:**
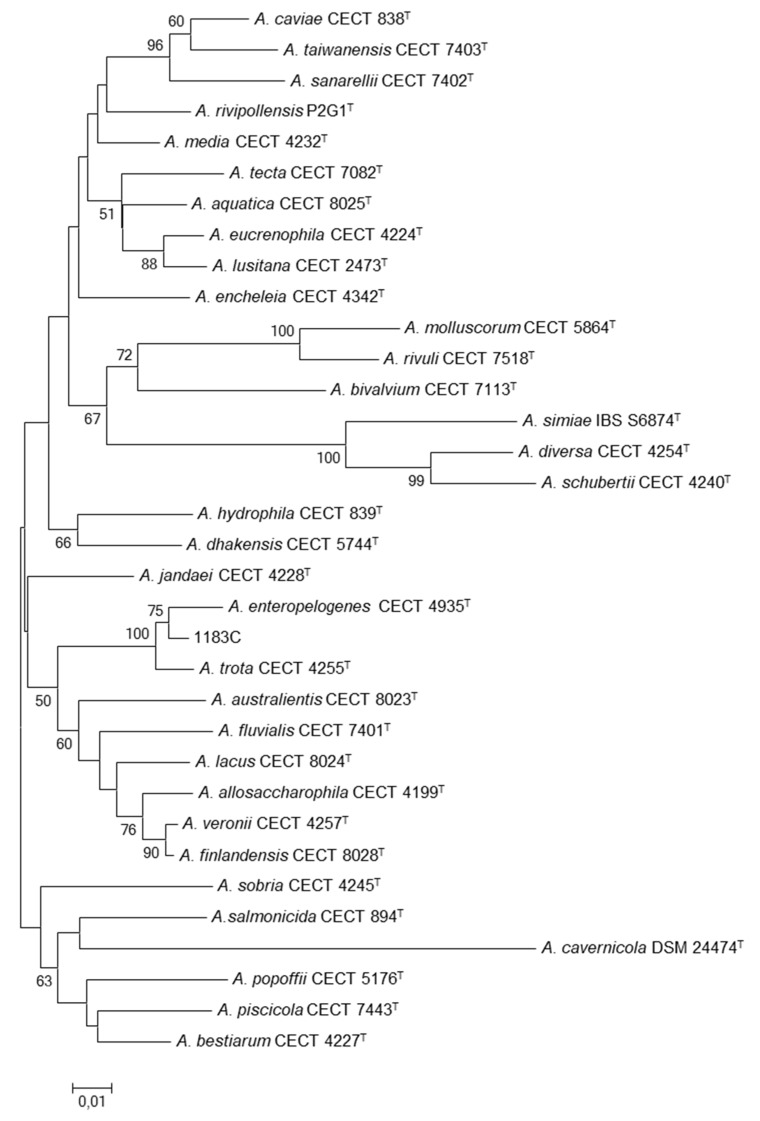
Phylogenetic tree based on *rpoD* gene (397 bp) with a neighbor-joining (NJ) algorithm. Numbers at nodes indicate bootstrap values (percentage of 1000 replicates). Bar 0.01 estimated nucleotide substitutions per site.

The isolate 1183C was sent to the Unit of Microbiology at the University Rovira i Virgili for re-identification by using the sequences of *rpoD* gene, as it was carried out routinely for all isolates identified as *Aeromonas* sp. at the hospital. The DNA extraction, amplification and sequencing were performed by using primers and conditions previously described [10]. A BlastN analysis with the obtained *rpoD* sequence revealed 99% similarity with a strain of *A. enteropelogenes.* Likewise, the phylogenetic tree constructed with the *rpoD* gene of the strain 1183C and the sequences of the type strains of all the *Aeromonas* spp. with a neighbor-joining (NJ) algorithm revealed that the sequence of the isolated strain clustered with the sequences of the type strains of *A. enteropelogenes* and *A. trota*, demonstrating that the strain belongs to these species; as indicated before, they are synonyms (Figure 1).

## 3. Discussion

Species of the genus *Aeromonas* are considered opportunistic emerging pathogens that cause diarrhea, bacteremia and wound infections [1,2]. This bacterium is mainly an enteric pathogen that affects with higher frequency in children, elderly people and immunocompromised individuals. The incidences of diarrhea caused by *Aeromonas* in children range from 2% to 13%, and in adults from 2% to 7% when the individuals are immunocompetent, however, it rises to 13% in immunocompromised individuals [2]. The species *A. trota,* has been isolated in association with diarrhea with a prevalence of 0.27% and it is considered a rare species [2,6,7,12]. Other data that support the enteropathogenicity of *A. trota* is the capacity to develop diarrhea in a healthy laboratory worker after an accidental ingestion of a pure culture suspension or the result of an experimental infection in a murine animal model [4,13]. Nevertheless, the descriptions of cases associated with *A. trota* are rare. The first case report published due to *A. trota* associated with diarrhea after the species description in 1991 dates back to 1996, and corresponded to a three-year-old boy that presented a mucous diarrhea not associated with other clinical manifestations [12]. Our case report is important since it represents, according to our knowledge, the second case of diarrhea due to *A. trota,* and it is the first one reported in adults.

Previous studies described *A. trota* as a rare species, being, as mentioned before, the only species of the genus susceptible to ampicillin [6,8,14,15,16,17]. Additionally, it has been described as resistant to cephalothin [6,14]. The strain isolated from our patient showed a similar resistance pattern to ampicillin and cephalothin.

In 1993, Collins et al. [7] demonstrated, based on the 16S rRNA gene sequence analysis, that *A. trota* [6] and *A. enteropelogenes* [9] were identical, with a similarity of 100% between the sequences of the type strains. The first species was isolated from feces collected in south-eastern Asia and the second was isolated from human feces in India. In addition, Huys et al. [8] confirmed with the DNA–DNA hybridization studies, as well as with phenotypic data that these strains represented the same species. The DNA–DNA hybridization values between the strains of the two species were 81%–99%, clearly above the 70% cut-off established to delimit different species, showing values of 40%–49% when comparing these two species with other non-related species such as *A. caviae* and *A. sobria* [8]. In addition, none of the 60 different phenotypical tests enabled the discrimination of the type strain of *A. trota* and *A. enteropelogenes* which also showed the same antibiotic susceptibility pattern [8].

On the basis of the Judicial Commission, *A. enteropelogenes* has nomenclatural priority, since this species was previously described [6,9]. The name *A. enteropelogenes* was included in the Validation List no. 38 [9], while the name of *A. trota* was announced in the Validation List no. 40 [6]. However, *A. trota* has been more used by the scientific community [8,18,19,20]. A recent PubMed search (04/21/2019) using “*Aeromonas trota*” yielded 55 citations, while a similar request using “*Aeromonas enteropelogenes*” produced only 20 records. A Request for an Opinion is necessary to change the nomenclature.

The MALDI-TOF is a fast and useful tool employed in many hospitals for the fast identification of bacteria, including those of the genus *Aeromonas* and it was shown to be more precise than the phenotypic methods [21,22]. The validation of MALDI-TOF was carried out by comparing the obtained results with those of molecular reference methods for the *Aeromonas* identification, such as the sequences of housekeeping genes [21,22]. The fact that the Biotyper database has few representatives of just a single representative of each species can hinder the correct identification of the species [21,22]. *Aeromonas* have changed continuously with the description of new taxa and reclassifications. Synonyms, such as “*Aeromonas punctata*” for *A. caviae*, “*A. trota*” for *A. enteropelogenes* and “*Aeromonas ichthiosmia*” for *A. veronii* are examples. However, the names of species not used, such as *A. ichthiosmia, A. punctata* or *A. enteropelogenes* are included in the Biotyper database [23] in parallel with the correct names without advising that they are synonyms. This is a problem in the clinical field because the clinicians do not work with taxonomy and might think that these synonyms represent different species. Based on these observations, we believe that it is important that the Biotyper database updates the taxonomic information indicating that the species *A. enteropelogenes*, is similar to *A. trota*.

## 4. Conclusion

The study intends to alert clinicians that the recognition of an *Aeromonas* strain susceptible to ampicillin may represent a strain of *A. trota* and advise a need for updating the MALDI-TOF database indicating that the species *A. enteropelogenes* is similar to *A. trota*.

## Figures and Tables

**Table 1:** Results obtained with MALDI-TOF Biotyper in two independent laboratories with different versions of the Biotyper database (V5 and V4).

Species/Strain	V5 Score	V4 Score
*A. enteropelogenes* DSM9381	2.182	2.400
*A. enteropelogenes* DSM7312	2.118	2.360
*A. enteropelogenes* DSM6394^T^	2.049	2.170
*A. jandaei* CECT4228^T^	0.035	1.991

**Table 2:** Antibiotic resistance pattern determined with the MicroScan WalkAway.

Antimicrobial agents	Result
Penicillins ^a^	Susceptible
Quinolones	Susceptible
Carbapenems	Susceptible
Amynoglycosides	Susceptible
Cephalosporins ^b^	Susceptible

^a^ In combination with beta-lactamase inhibitors; ^b^ Except cephalothin.
